# 7-Day National Institutes of Health Stroke Scale as a surrogate marker predicting ischemic stroke patients’ outcome following endovascular therapy

**DOI:** 10.1515/tnsci-2022-0307

**Published:** 2023-10-19

**Authors:** Yuzheng Lai, Eric Jou, Mohammad Mofatteh, Thanh N. Nguyen, Jamie Sin Ying Ho, Francesco Diana, Adam A. Dmytriw, Jianfeng He, Wenshan Yan, Yiying Chen, Zile Yan, Hao Sun, Leonard L. Yeo, Yimin Chen, Sijie Zhou

**Affiliations:** Department of Neurology, Guangdong Provincial Hospital of Integrated Traditional Chinese and Western Medicine, Foshan, Guangdong, China; Nanhai District Hospital of Traditional Chinese Medicine of Foshan City, Foshan, 528000, Guangdong, China; School of Clinical Medicine, University of Cambridge, Cambridge, United Kingdom; School of Medicine, Dentistry and Biomedical Sciences, Queen’s University Belfast, Belfast, United Kingdom; Department of Neurology, Radiology, Boston University Chobanian & Avedisian School of Medicine, Boston, MA, United States; Department of Medicine, Royal Free London NHS Foundation Trust, London, United Kingdom; Department of Neuroradiology, A.O.U. San Giovanni di Dio e Ruggi d’Aragona, University of Salerno, Salerno, Italy; Neuroendovascular Program, Massachusetts General Hospital, Harvard Medical School, Boston, MA, USA; Department of Neurology and Advanced National Stroke Center, Foshan Sanshui District People’s Hospital, Foshan, 528100, Guangdong, China; Division of Neurology, Department of Medicine, National University Health System, Singapore, Singapore; Department of Medicine, Yong Loo Lin School of Medicine, National University of Singapore, Singapore; Department of Neurology, Neuro International Collaboration (NIC), Foshan, China; Department of Surgery of Cerebrovascular Diseases, First People’s Hospital of Foshan, Foshan, 528000, Guangdong, China

**Keywords:** stroke, prognosis, mechanical thrombectomy, endovascular thrombectomy, NIHSS

## Abstract

**Background:**

Early neurological deterioration after endovascular thrombectomy (EVT) is associated with poor prognosis. National Institutes of Health Stroke Scale (NIHSS) score measured at 24 h after EVT may be a better outcome predictor than other methods that focus on changes in NIHSS. Nevertheless, clinical fluctuations in ischemic stroke patients during the immediate phase after symptoms onset are well recognized. Therefore, a delayed NIHSS evaluation may improve prognostic accuracy. We evaluate the 7-day NIHSS in predicting long-term patient outcomes after EVT.

**Methods:**

This was a multi-center retrospective cohort study of 300 consecutive ischemic stroke patients with large vessel occlusion who underwent EVT at three-stroke centers in China from August 2018 to March 2022. NIHSS was recorded on admission, pre-EVT, 24 h, and 7 days after EVT.

**Results:**

A total of 236 eligible patients were subdivided into two groups: 7-day NIHSS ≤6 and NIHSS >6 post-EVT. 88.29% achieved a favorable outcome (modified Rankin Scale 0–2) in the NIHSS ≤6 group compared to 15.20% in the NIHSS >6 group at 90 days, and an improved favorable outcome in the former group was observed after adjusting for potential confounding factors (adjusted odds ratio 39.7, 95% confidence interval, 17.5–89.7, *p* < 0.001).

**Conclusion:**

The 7-day NIHSS score may be a reliable predictor of 90-day stroke patient outcome after EVT.

## 1 Introduction

Mechanical endovascular thrombectomy (EVT) has been demonstrated to improve outcomes in patients with large vessel occlusion ischemic stroke when performed within 6 h of symptom onset or up to 24 h in selected patients [1], leading to its establishment as the current standard of care [2,3,4]. Identifying early prognostic markers that accurately predict long-term outcomes in patients after EVT is paramount in facilitating medical decision-making and can be utilized as surrogate endpoints in clinical trials [4].

Early neurological deterioration following EVT has been associated with poor 90-day outcomes [5], indicating that short-term neurological examinations may have value in predicting long-term patient prognosis. The National Institute of Health Stroke Scale (NIHSS) enables quantification of stroke severity in patients with a higher score, reflecting more impairment. Accordingly, early neurological recovery characterized by a greater than 2 point NIHSS improvement at 2 h or four points at 24 h is associated with good outcomes at 3 months [6], with other reports finding similar results [4,7]. Importantly, a recent study found an NIHSS of 8 or less at 24 h after EVT to be the best predictor of favorable outcomes compared to other methods that measured changes in NIHSS [8]. Clinical fluctuations in stroke patients, in particular during the immediate phase after symptom onset, are well recognized [9,10], and a delayed NIHSS evaluation may improve the accuracy of the neurological examination and provide early prognostication.

In this study, we evaluated the efficacy of the 7-day NIHSS in predicting long-term patient outcomes after EVT. To our knowledge, this is the first study to use 7-day NIHSS in post-EVT to predict ischemic patients’ outcomes.

## 2 Materials and methods

### 2.1 Patient selection

This is a retrospective analysis of prospectively collected data from consecutive ischemic stroke patients who underwent EVT from August 2018 to March 2022 at three academic comprehensive stroke centers in China. The data were derived from the Big Data Observatory Platform for stroke in China and from the hospital data platform.

Inclusion criteria were as follows: (1) patients who underwent emergency endovascular therapy, (2) age ≥18 years old, and (3) presented within 24 h of stroke onset. The exclusion criteria were as follows: (1) patients with pre-EVT NIHSS ≤5, (2) missing follow-up data, and (3) missing 7 ± 2-day NIHSS after EVT

### 2.2 Data collection

We collected the following data: age, sex, risk factors of cerebrovascular disease (hypertension, diabetes mellitus, coronary artery disease, atrial fibrillation (AF), prior stroke, hyperlipemia, coronary kidney disease, smoking status), initial premorbid modified Rankin Scale (mRS), The Alberta stroke program early CT score, door-to-needle time (DNT), onset-to-needle time (ONT), door to puncture time (DPT), last known normal-to-puncture time, door-to-recanalization time (DRT), and modified thrombolysis in cerebral infarction (mTICI) post-thrombectomy. Successful reperfusion was defined as mTICI ≥2b.

Attending neurologists or neurosurgeons measured and recorded the NIHSS of consecutive patients and entered the data into the database prospectively. NIHSS was recorded on admission, pre-EVT, 24 h after EVT, and 7 days after EVT.

### 2.3 Outcome measures

The primary outcome was mRS 3 months after EVT. The follow-up mRS was measured routinely by specialized stroke nurses and doctors by telephone or in-person appointments during outpatient follow-up. A favorable outcome was defined as mRS of 0–2 at 90 days. Walking independence was defined as mRS of 3. The secondary outcome was 90-day mortality.

### 2.4 Statistical analysis

Patients with 7-day NIHSS >6 were compared to those with NIHSS ≤6. The non-parametric Mann–Whitney *U* test was performed using the IBM SPSS version 26 (IBM-Armonk, NY) to analyze non-normally distributed continuous data, reported as medians along with the interquartile range. Normally distributed data were reported as means with corresponding standard deviations (SD) and compared using the student’s *t*-test. Results were considered statistically significant if the *p*-value was less than 0.05.


**Ethical approval:** The research related to human use has been complied with all the relevant national regulations, institutional policies and in accordance the tenets of the Helsinki Declaration, and has been approved by the authors’ institutional review board or equivalent committee. The study was approved by The Foshan Sanshui District People’s Hospital review board (No. SRY-EC-2022-051) and the Guangdong Provincial Hospital of Integrated Traditional Chinese and Western Medicine Hospital review board (No. 2022-085).
**Informed consent:** Informed consent has been obtained from all individuals included in this study.

## 3 Results

In total, 300 EVT patients were evaluated. There were four patients excluded for missing follow-up data, 58 patients excluded due to death or discharge before seven days, thus missing 7-day NIHSS, and another two patients excluded for NIHSS pre-EVT of less than 6. This resulted in a cohort of 236 patients. The patients were divided into two groups according to 7-day NIHSS ≤6 (*n* = 111) and NIHSS >6 after EVT (*n* = 125). The baseline characteristics of patients are demonstrated in Table 1.

**Table 1:** Comparison of baseline characteristics of patients with NIHSS ≤6 and NIHSS >6 on day 7

	NIHSS ≤6	NIHSS >6	*X* ^2^/*t*/*z*	*P*
Number	111	125		
Age, mean ± SD	61.69 ± 13.33	66.88 ± 12.18	3.124	0.002
Male, *n* (%)	73 (65.77)	89 (71.20)	0.807	0.369
Hypertension, *n* (%)	59 (53.15)	82 (65.60)	3.787	0.052
Diabetes mellitus, *n* (%)	16 (14.41)	32 (25.60)	4.540	0.033
CAD, *n* (%)	14 (12.61)	25 (20.00)	2.326	0.127
AF, *n* (%)	38 (34.23)	45 (36.00)	0.080	0.777
Prior stroke, *n* (%)	20 (18.02)	28 (22.40)	0.697	0.404
Hyperlipemia, *n* (%)	24 (21.62)	18 (14.40)	2.096	0.148
CKD, *n* (%)	8 (7.21)	9 (7.20)	0.001	0.998
Smoker, *n* (%)	26 (23.42)	23 (18.40)	0.902	0.342
NIHSS pre-EVT (IQR)	13.00 (10.00, 17.00)	16.00 (12.00, 20.00)	−4.116	<0.001
ASPECTS pre-treatment (IQR)	8.00 (8.00, 9.00)	8.00 (8.00, 9.00)	−0.967	0.334
mRS pre-premorbid (IQR)	0.00 (0.00, 0.00)	0.00 (0.00, 0.00)	−1.679	0.093
Occluded vessel				
Distal/terminal ICA, *n* (%)	15 (13.51)	26 (20.80)	6.833	0.337
MCA-M1, *n* (%)	57 (51.35)	48 (38.40)		
MCA-M2, *n* (%)	7 (6.31)	8 (6.40)		
MCA-M3, *n* (%)	0 (0.00)	1 (0.80)		
Tandem, *n* (%)	16 (14.41)	15 (12.00)		
Basilar, *n* (%)	12 (10.81)	20 (16.00)		
Others, *n* (%)	4 (3.60)	7 (5.60)		
IV thrombolysis, *n* (%)	50 (45.05)	39 (31.20)	4.798	0.028
DNT	41.00 (28.50, 51.50)	45.00 (33.00, 65.00)	−1.434	0.152
ONT	129.00 (95.00, 178.50)	113.00 (89.00, 146.00)	−1.063	0.288
Median DPT (IQR), min	126.00 (100.00, 190.00)	150.00 (113.50, 205.00)	−2.156	0.031
Median DRT (IQR), min	205.00 (153.00, 258.00)	245.00 (186.50, 311.00)	−3.113	0.002
Median PRT (IQR), min	53.00 (35.00, 80.00)	73.00 (47.50, 110.00)	−3.585	<0.001
Median LKNPT (IQR), min	290.00 (195.00, 446.00)	305.00 (190.00, 480.50)	−0.731	0.465
mTICI post ≥2*b*, *n* (%)	23 (82.14)	102 (49.04)	10.856	0.001
sICH, *n* (%)	0 (0.00)	16 (12.80)	15.241	<0.001

Abbreviations: AF; atrial fibrillation; ASPECTS, The Alberta stroke program early CT score; CE, cardioembolic; CKD, chronic kidney disease; CAD, coronary artery disease; DM, diabetes mellitus; DPT, door-to-puncture time; DRT, door-to-recanalization time; EVT, endovascular thrombectomy; IQR, interquartile range; IV, intravenous; LKNPT, last-known normal-to-puncture time; mTICI, modified thrombolysis in cerebral infarction; NIHSS, National Institutes of Health Stroke Scale; sICH, symptomatic intracranial hemorrhage.

Compared to the 7-day NIHSS >6 group, the NIHSS ≤6 group was younger (61.69, SD 13.33 years vs 66.88, SD 12.18 years, *p* = 0.002) and had a lower prevalence of diabetes mellitus (14.41 vs 25.60%, *p* = 0.03) and lower median NIHSS pre-EVT (13.00 vs 16.00, *p* < 0.001). There were no significant differences in sex, coronary artery disease (CAD), AF, prior stroke, hypertension, hyperlipidemia, smoking history, and occluded vessels. Compared to the NIHSS >6 group, a greater number of patients in the NIHSS ≤6 group received IV thrombolysis (45.05 vs 31.20%, *p* = 0.028) (Table 1).

In terms of the time metrics of EVT, patients with 7-day NIHSS ≤6 had significantly shorter median DPT (126.00 vs 150.00 min, *p* = 0.031), median DRT (205.00 vs 245.00 min, *p* = 0.002), and median PRT (53.00 vs 73.00 min, *p <* 0.001) than patients with NIHSS >6. In addition, none of the NIHSS ≤6 group had symptomatic intracerebral hemorrhage (sICH), compared to 12.80% of the NIHSS >6 group (*p* < 0.001) (Table 1).

### 3.1 Primary outcome: 90-day mRS

The median mRS discharge was lower in the NIHSS ≤6 group compared to those with NIHSS >6 (1.00 vs 4.00, *p <*0.001) (Table 2).

**Table 2:** Comparison of 90-day clinical outcomes in patients with 7-day NIHSS ≤6 compared to NIHSS ＞6

	7-day NIHSS ≤6	7-day NIHSS >6	*X* ^2^/*z*	*p*
Median mRS discharge (IQR)	1.00 (1.00, 2.00)	4.00 (4.00, 5.00)	−11.33	<0.001
90-Day favorable outcome, *n* (%)	98 (88.29)	19 (15.20)	125.63	<0.001
90-Day mortality, *n* (%)	4 (3.60)	32 (25.60)	22.01	<0.001

Favorable outcome: mRS (0–2) at 3 months. Poor outcome: mRS ≥3. Abbreviations: IQR, interquartile range; mRS, modified Rankin scale; NIHSS, National Institutes of Health Stroke Scale.

At 90 days, 88.29% achieved a favorable outcome (mRS 0–2) in the NIHSS ≤6 group while only 15.20% of patients achieved a 90-day favorable outcome in the NIHSS >6 group (*p* < 0.001). Favorable outcome at 90 days remained significantly higher in patients with NIHSS ≤6 compared to NIHSS >6 even after adjustment for age, hypertension, diabetes mellitus, pre-EVT NIHSS, and pre-morbid mRS (adjusted odds ratio [OR] 39.7, 95% confidence interval [CI] 17.5–89.7, *p* < 0.001). The adjusted analysis of 90-day outcomes is shown in Tables 3 and 4.

**Table 3:** Comparison of 90-day outcomes of patients with 7-day NIHSS ≤6 versus NIHSS >6 after adjusting for baseline relevant factors

	Adjusted OR*	95% CI	*p*
90-Day favorable outcome (mRS 0–2), *n* (%)	39.666	17.548–89.662	<0.001
90-Day poor outcome (mRS ≥3), *n* (%)	0.025	0.011–0.057	<0.001
90-Day mortality, *n* (%)	0.156	0.051–0.474	<0.001

Model: *Adjusted for age, hypertension, diabetes, pre-EVT NIHSS, and pre-morbid mRS. Abbreviation: mRS, modified Rankin scale.

**Table 4:** Comparison of 90-day outcomes of patients with 7-day NIHSS ≤6 versus NIHSS >6 after adjusting for relevant factors

	Adjusted OR*	95% CI	*p*
90-Day favorable outcome (mRS 0–2), *n* (%)	33.822	13.834–82.685	<0.001
90-Day poor outcome (mRS ≥3), *n* (%)	0.030	0.012–0.072	<0.001
90-Day mortality, *n* (%)	0.223	0.067–0.740	0.014

Model: *Adjusted for age, hypertension, diabetes, pre-EVT NIHSS and pre-morbid mRS, MCA-M2, IV thrombolysis, DRT, sICH, mTICI post ≥2b. Abbreviation: mRS, modified Rankin scale.

**Figure 1:**
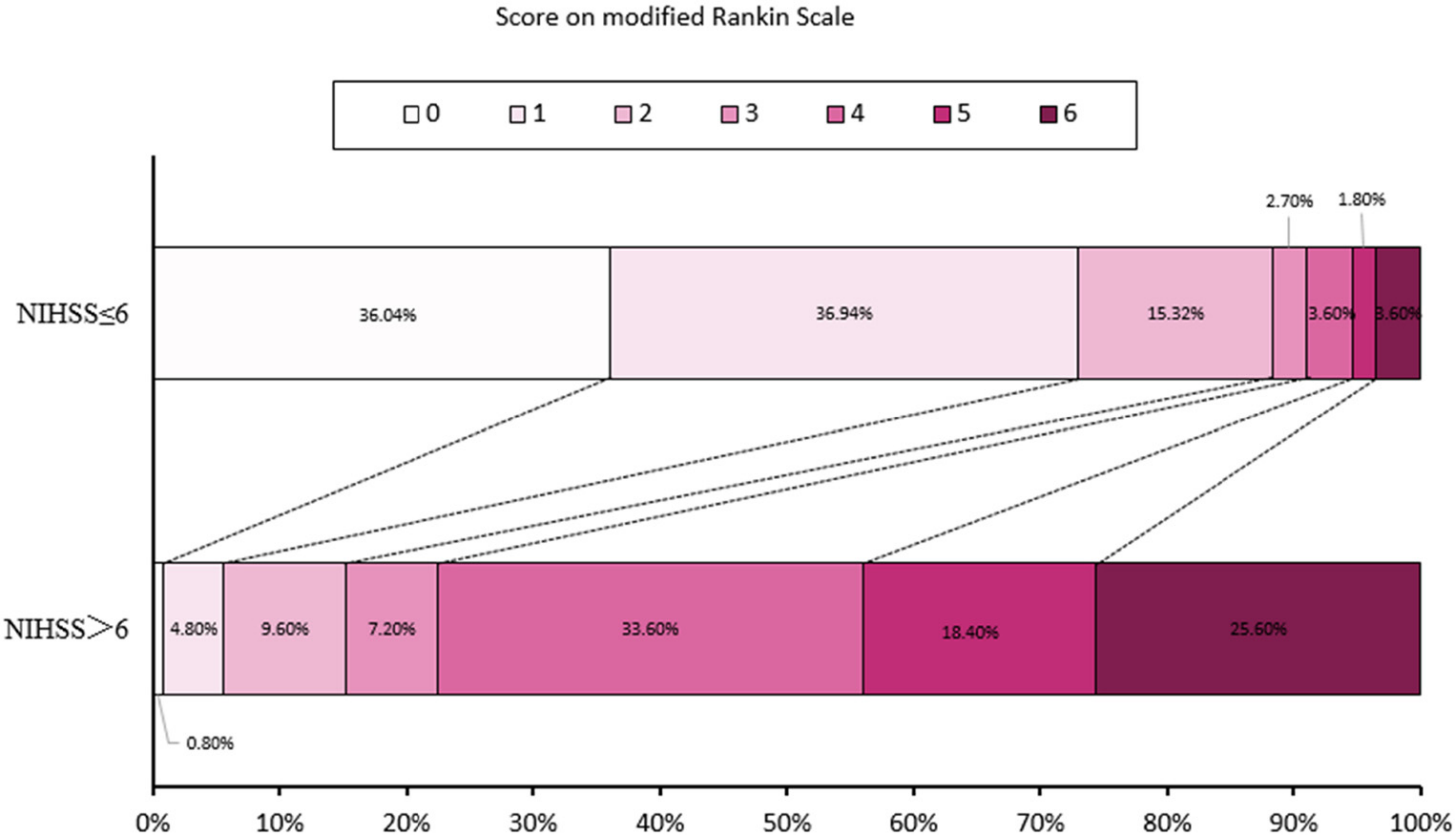
Distribution of 90-day mRS in patients with NIHSS ≤6 and NIHSS >6 on day 7.

The distribution of 90-day mRS in patients with NIHSS ≤6 and NIHSS >6 on day 7 is shown in Figure 1 and Table S1.

**Figure 2:**
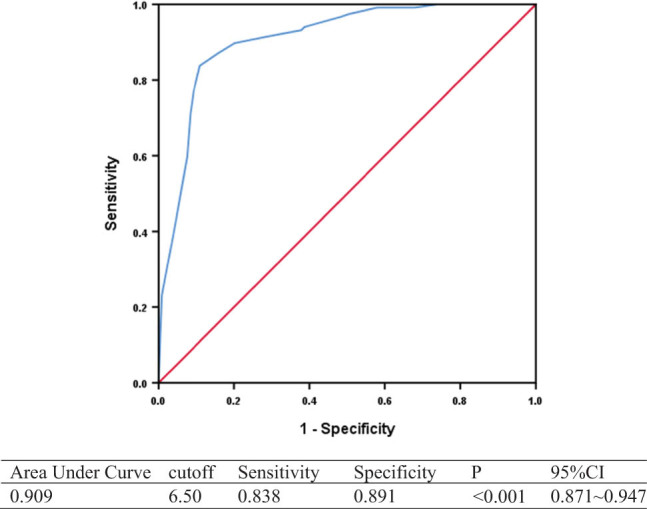
ROC of 7-day NIHSS predicting 90-day favorable outcome (mRS 0–2).

The optimal cut-off of 7-day NIHSS 6.50 had a specificity of 0.891 and a sensitivity of 0.838 for the prediction of favorable functional outcome at 90 days (area under the curve [AUC] 0.909, 95% CI 0.871–0.947, *p* ＜0.001) (Figure 2).

### 3.2 Secondary outcome: 90-day mortality

**Figure 3:**
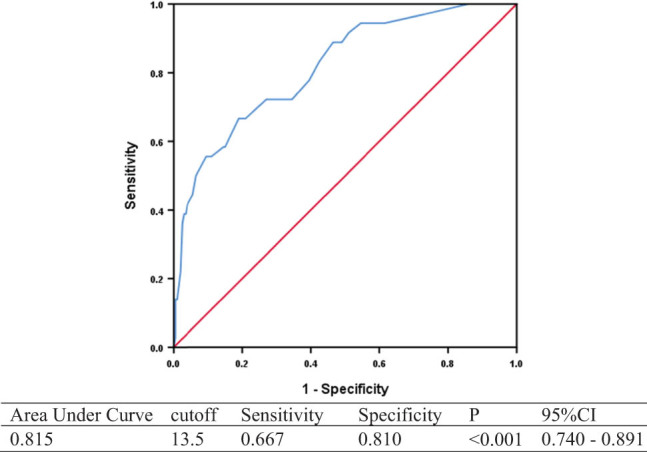
ROC of 7-day NIHSS predicting 90-day mortality.

Mortality at 90 days was significantly lower in patients with 7-day NIHSS ≤6 compared to NIHSS >6 on univariable analysis (25.60 vs 3.60%, *p* < 0.001) (Table 2) and on adjusted analysis (adjusted OR 0.156, 95% CI 0.051–0.474, *p* = 0.001) (Table 3). The optimal cut-off of 7-day NIHSS 13.5 had a specificity of 0.810 and a sensitivity of 0.667 for the prediction of mortality at 90 days (AUC 0.815, 95% CI 0.740–0.891, *p* ＜0.001) (Figure 3).

**Figure 4:**
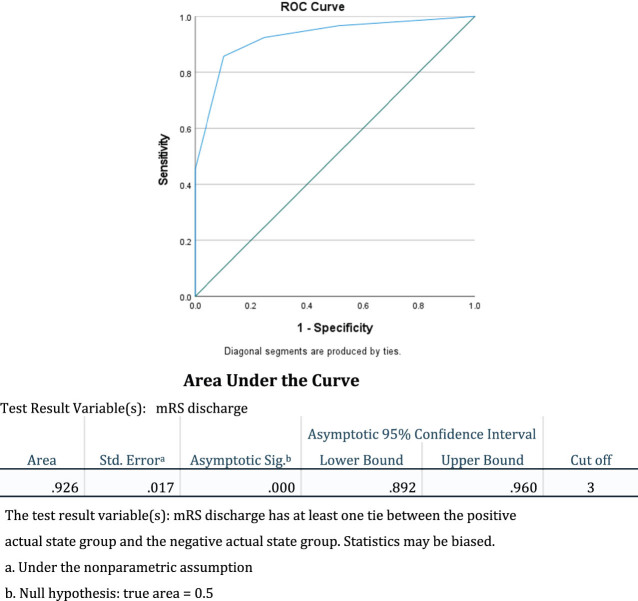
ROC of discharge mRS.

Furthermore, we calculated the mRS at discharge as shown in Figure 4.

## 4 Discussion

Our study found that NIHSS on day 7 is a predictor of 90-day clinical outcomes post-EVT, including favorable functional outcomes on mRS and mortality.

Stroke severity measured by baseline NIHSS on presentation is well established to be predictive of outcomes in ischemic stroke [11]. As such, the 2019 American Heart Association/American Stroke Association guidelines recommend that baseline NIHSS should be assessed, and stroke outcomes measures are strongly influenced by baseline stroke severity on NIHSS [12]. Given the strong association of NIHSS with 90-day outcomes, change in NIHSS or early NIHSS at 24 h has become an attractive surrogate for outcomes in clinical trials, particularly in patients who may be considered non-compliant with long-term follow-up. For example, the Blood Pressure after Endovascular Therapy for Ischemic Stroke study and the randomized controlled trial by the National Institute of Neurological Disorders and Stroke rt-PA Stroke Study Group used a 24-h change in NIHSS as a surrogate outcome for early neurological recovery [13,14]. However, the optimal surrogate markers for early neurological recovery are not established, and a post hoc analysis of a prospective cohort study on EVT-treated patients at 12 stroke centers in the US found that the absolute 24-h NIHSS adjusted for baseline NIHSS on multivariable analysis had the highest predictive power compared to 24-h change in NIHSS and percentage change in NIHSS [7]. The 24-h NIHSS of ≤7 had a sensitivity of 80.1% and a specificity of 80.4% for predicting 90-day mRS score of 0–2 [7], but the predictive value of NIHSS scores beyond 24 h in post-EVT stroke patients requires further study.

Consistent with multiple studies that have reported that 7-day NIHSS is a sensitive measure for acute stroke outcomes [15,16,17], we found in our study that the 7-day NIHSS of >6 predicted 90-day mRS 0–2 with a sensitivity of 83.8% and a specificity of 89.1%. This is comparable to a previous study on serial NIHSS post-acute symptomatic middle cerebral artery territory infarcts, which found that 7-day NIHSS of ≥6 predicted poor outcomes with a sensitivity of 84% and a specificity of 94%, and had the highest AUC ROC for poor outcomes among baseline, day 3, and day 14 NIHSS scores [18]. In a random sample of 10,000 patients who underwent recombinant tissue-type plasminogen activator (rtPA), out of 4 clinical endpoints of mRS at 30 and 90 days, and NIHSS at 7 and 90 days, 7-day NIHSS required the smallest sample size to generate statistical power of >80% to detect a treatment effect of alteplase [15] and may be a sensitive endpoint for clinical trials. We also observed that 7-day NIHSS was predictive of 90-day mortality, after adjustment for baseline NIHSS, age, hypertension, diabetes, and pre-morbid mRS, and a cut-off of 13.5 had a specificity of 81.0% and a sensitivity of 66.7%. Therefore, the 7-day NIHSS may be a feasible and sensitive surrogate marker of outcomes post-EVT, such as 90-day mRS and mortality, but further validation in prospective studies and RCTs is needed.

Previous studies have shown that age is a significant risk factor for the outcome of stroke patients [19–21]. Patients in the NIHSS >6 group tended to be older, compared to the NIHSS ≤6 group (*p* = 0.002), which can be considered a confounding factor. To remove the possibility of such confounding factors influencing our conclusion, we demonstrated that the reported favorable outcome at 90 days remained significantly higher even after adjustment for age in this cohort.

The NIHSS quantifies the extent of neurological deficits present after acute ischemic stroke and represents the severity of stroke. While baseline NIHSS identifies patients presenting with severe strokes, NIHSS after 1 week can identify a subset of patients with poor neurological recovery, which may be more strongly associated with longer-term outcomes. Early neurological recovery, measured by improvement in NIHSS by 10 points or absolute score of ≤4 within 2 h (OR 2.4), and continuous neurological recovery, or improvement in NIHSS by 8 points at 2–24 h (OR 7.3), were independent predictors of good functional outcomes at 3 months in a prospective cohort study [14]. The recovery process post-stroke is time dependent, and the most significant improvements occur within the first 3 months when surviving neurons in peri-infarct areas enlarge their dendritic trees and sprout new axons to form new synaptic connections in local and distant brain areas [22,23]. Therefore, the 7-day NIHSS may better predict the efficacy of this neurological recovery process compared to baseline NIHSS and hence serve as a better surrogate for functional recovery post-stroke. Furthermore, workflow optimization and identifying parameters, such as recanalization status, can play significant roles in shortening DNT and DPT, thus improving patient outcomes and reducing mortality [24–26].

There are several limitations to this study. First, this is a small-to-moderate size retrospective cohort study and hence may be susceptible to confounding factors; therefore, a causative relationship cannot be determined. However, we have adjusted for clinical factors most predictive of functional outcomes, such as age, hypertension, diabetes, and baseline NIHSS [27]. The biological feasibility and temporal association of 7-day NIHSS and 90-day functional outcome also increases the reliability of the observed relationship. Due to the lack of 7-day NIHSS in patients who died or were discharged before 7 days, there may be a selection bias in the cohort included in our study, as the extremes of patient presentations were excluded (those with very severe presentation who died before 7 days, and those with very mild presentation who were discharged before 7 days). In addition, patients with basilar occlusion were included in the study with 20 basilar occlusions in the NIHSS >6 group versus 12 in the NIHSS ≤6 group. Although there were no statistically significant differences (*p* = 0.245) between the two groups in our study, and previous studies have demonstrated the effectiveness of EVT in basilar artery occlusion [28,29], such variations may serve as an additional cofounder which needs to be addressed in future studies. Exclusion criteria resulted in the removal of 64 patients (21.3%) from the final analysis. This is a substantial number, and future large-scale studies are required to address such limitations. Despite such limitations, these results can prompt further studies at other stroke centers. Further large prospective studies and RCTs are needed to validate the use of 7-day NIHSS to predict outcomes post-EVT and its use as a surrogate marker in clinical trials. Compared to 90-day outcomes that require longer-term follow-up and are more resource intensive, shorter-term outcomes like 7-day NIHSS may have the potential to be both cost-effective and pragmatic, but this needs to be investigated in further studies.

## 5 Conclusions

In this retrospective analysis of a prospectively maintained database, a 7-day NIHSS score of ≤6 was a predictor of 90-day functional outcomes (mRS 0–2) and mortality. Further prospective studies and RCTs are needed to validate the use of 7-day NIHSS to predict outcomes post-EVT and as a pragmatic, surrogate marker in clinical trials.

## Supplementary Material

Supplementary Table
