# Supplementary material for: 7-Day National Institutes of Health Stroke Scale as a surrogate marker predicting ischemic stroke patients’ outcome following endovascular therapy
Source: Transl Neurosci. 2023 Oct 19;14(1):20220307. doi: 10.1515/tnsci-2022-0307 (PMC10590605; doi:10.1515/tnsci-2022-0307)
Supplement: Supplementary Table [file tnsci-2022-0307-sm.pdf]

Supplementary material

Table S1: 90-day mRS in patients with 7-day NIHSS ≤ 6 versus NIHSS > 6

	**NIHSS ≤ 6**	**NIHSS > 6**	X^2	***P***
mRS 0, n, %	40 (36.04)	1 (0.80)	137.486	< 0.001
mRS 1, n, %	41 (36.94)	6 (4.80)		
mRS 2, n, %	17 (15.32)	12 (9.60)		
mRS 3, n, %	3 (2.70)	9 (7.20)		
mRS 4, n, %	4 (3.60)	42 (33.60)		
mRS 5, n, %	2 (1.80)	23 (18.40)		
mRS 6, n, %	4 (3.60)	32 (25.60)		

NIHSS, National Institutes of Health Stroke Scale.
